# Adherence to newer second‐line oral antidiabetic drugs among people with type 2 diabetes—A systematic review

**DOI:** 10.1002/prp2.1185

**Published:** 2024-03-07

**Authors:** Nynne Sophie Holdt‐Caspersen, Claus Dethlefsen, Peter Vestergaard, Ole Hejlesen, Stine Hangaard, Morten Hasselstrøm Jensen

**Affiliations:** ^1^ Department of Biostatistics Novo Nordisk Aalborg Denmark; ^2^ Department of Health Science and Technology Aalborg University Aalborg Denmark; ^3^ Department of Mathematical Sciences Aalborg University Aalborg Denmark; ^4^ Steno Diabetes Center North Denmark Aalborg University Hospital Aalborg Denmark; ^5^ Department of Endocrinology Aalborg University Hospital Aalborg Denmark; ^6^ Department of Clinical Medicine Aalborg University Aalborg Denmark; ^7^ Department of Data Orchestration Novo Nordisk Søborg Denmark

**Keywords:** adherence, oral antidiabetic drug, review, type 2 diabetes

## Abstract

The adherence to oral antidiabetic drugs (OADs) among people with type 2 diabetes (T2D) is suboptimal. However, new OADs have been marketed within the last 10 years. As these new drugs differ in mechanism of action, treatment complexity, and side effects, they may influence adherence. Thus, the aim of this study was to assess the adherence to newer second‐line OADs, defined as drugs marketed in 2012–2022, among people with T2D. A systematic review was performed in CINAHL, Cochrane Trials, Embase, PubMed, PsycINFO, and Scopus. Articles were included if they were original research of adherence to newer second‐line OADs and reported objective adherence quantification. The quality of the articles was assessed using JBI's critical appraisal tools. The overall findings were reported according to the preferred reporting items for systematic reviews and meta‐analyses (PRISMA) guidelines and summarized in a narrative synthesis. All seven included articles were European retrospective cohort studies investigating alogliptin, canagliflozin, dapagliflozin, empagliflozin, and unspecified types of SGLT2i. Treatment discontinuation and medication possession ratio (MPR) were the most frequently reported adherence quantification measures. Within the first 12 months of treatment, 29%–44% of subjects on SGLT2i discontinued the treatment. In terms of MPR, 61.7%–94.9% of subjects on either alogliptin, canagliflozin, dapagliflozin, empagliflozin or an unspecified SGLT2i were adherent. The two investigated adherence quantification measures, treatment discontinuation and MPR, suggest that adherence to the newer second‐line OADs may be better than that of older OADs. However, a study directly comparing older and newer OADs should be done to verify this.

## Graphical abstract

The adherence to newer second‐line oral antidiabetic drugs among people with type 2 diabetes was investigated in this study. A systematic literature search was performed in six databases. A total of 14948 records was retrieved from the databases of which 7 articles were included. The reported medication possession ratio (MPR) and treatment discontinuation was extracted from the articles, and these findings were visualised in figures and summarized in a narrative synthesis. The findings indicate that the adherence to the newer second‐line OADs may be better than that of older OADs, but a study directly comparing older and newer OADs should be performed to verify this.

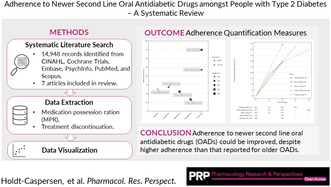

**Abbreviations**
ADA: American Diabetes Association
DPP‐4i: Dipeptidyl peptidase 4 inhibitor
EASD: European Association for the Study of Diabetes
EMA: European Medicines Agency
GLP‐1 RA: Glucagon‐like peptide 1 receptor agonist
MPR: Medication possession ratio
OAD: Oral antidiabetic drug
SGLT2i: Sodium‐glucose cotransporter 2 inhibitor
SU: Sulfonylurea
T2D: Type 2 diabetes
TZD: Thiazolidinedione

## 1 INTRODUCTION

In 2021, approximately 536 million people aged 20–79 had a diagnosis of diabetes. The vast majority of these people had a diagnosis of type 2 diabetes (T2D).^1^ T2D is characterized by a dysregulation of the blood glucose, resulting in hyperglycemia, which over time may lead to long‐term complications such as cardiovascular disease, nephropathy, and neuropathy. Therefore, the aim of the antidiabetic treatment is to keep the blood glucose within the normal range.^2^ The recommended first‐line treatment, according to the American Diabetes Association (ADA) and the European Association for the Study of Diabetes (EASD), is lifestyle modification combined with metformin treatment.^2^ When the first‐line treatment fails to keep the blood glucose within the normal range due to disease progression, a second‐line drug is added to the treatment regimen.^2^ The recommended second‐line treatment is either a sulfonylurea (SU), thiazolidinedione (TZD), dipeptidyl peptidase 4 inhibitor (DPP‐4i), glucagon‐like peptide 1 receptor agonist (GLP‐1 RA), sodium‐glucose cotransporter 2 inhibitor (SGLT2i) or basal insulin.^2^

Potential comorbidities and the patient's preferences should be considered when choosing which second‐line antidiabetic drug to add to the treatment regimen.^2^ The patient's preference regarding the route of administration, side effects, and medication complexity may influence the patient's treatment adherence, as these are all potential barriers to adherence.^3^, ^4^, ^5^ As all five recommended non‐insulin second‐line antidiabetic drug classes are available in an oral form, it is possible for most patients with T2D to have a treatment regimen consisting entirely of oral antidiabetic drugs (OADs).

The adherence to OADs among people with T2D has previously been investigated using objective adherence quantification measures and reported in systematic reviews and meta‐analysis.^6^, ^7^, ^8^, ^9^, ^10^ Cramer,^6^ Krass et al.,^7^ Iglay et al.,^8^ and Evans et al.^10^ all found that the adherence to OADs is suboptimal. Furthermore, Evans et al.^10^ and McGovern et al.^9^ found that the adherence between drug classes varied significantly. In 2012, the first drug of an entirely new drug class, the SGLT2is, was approved by the European Medicines Agency (EMA).^11^, ^12^ Since 2012, several new OADs, used as second‐line treatment, have been marketed. These are the SGLT2is canagliflozin, dapagliflozin, empagliflozin, and ertugliflozin, the DPP‐4i alogliptin, and the GLP‐1 RA oral semaglutide.^13^ These new drugs differ in mechanism of action, treatment complexity, and side effects, which all may influence the adherence. The aim of this study was to assess the adherence to newer second‐line OADs, defined as drugs marketed within the last 10 years (2012–2022), among people with T2D.

## 2 MATERIALS AND METHODS

A systematic literature search was performed to identify studies, which investigated the adherence to newer second‐line OADs. The search was performed on the 12 and 13 July 2022 and included articles published from 2012 to the search date. The protocol of the systematic literature search was preregistered on PROSPERO (registration ID: CRD42022344503).

### 2.1 Search strategy

The literature search was performed as a block search consisting of the three blocks “T2D”, “OAD”, and “Adherence”. Each block consisted of synonyms, near‐synonyms, acronyms, and relevant index terms. All terms were searched for in the title and abstract of the articles as free text. The literature search was performed in six databases: CINAHL, Cochrane Trials, Embase, PubMed, PsycINFO, and Scopus. The search string was adapted to each database (the full search string of each database is shown in Data S1). Furthermore, the search was restricted to articles written in English language only.

### 2.2 Study selection

The articles retrieved from each database were pooled and duplicates were removed. Following, articles were screened based on the title and abstract, and the remaining articles underwent full‐text screening. Articles were included if (1) the study subjects received metformin and one type of a newer second‐line OAD, (2) the study investigated adherence to a newer second‐line OAD, (3) the article was available in full text, and (4) the article was original research. Studies focusing on subjects with comorbidities or were set during special circumstances were excluded from the study, thereby obtaining a relatively homogeneous study group. Moreover, as subjects with comorbidities in general take more medication and the choice of antidiabetic medication may be conditioned by recommendations, adherence may be influenced. Likewise, the subjects' behavior regarding treatment adherence might be different during special circumstances compared to everyday settings and could potentially also influence adherence. These potential confounding variables were minimized by excluding articles if (1) the study subjects were younger than 18 years, (2) the study subjects had a diagnosis of diabetes other than T2D, (3) the inclusion criteria of the study included comorbidities, and (4) the study was set during special circumstances, such as Ramadan or the COVID‐19 pandemic. The selection of studies was conducted and reported according to the preferred reporting items for systematic reviews and meta‐analyses (PRISMA)^14^ using the software Rayyan, developed by Ouzzani et al.,^15^ to record the decision for each article.

### 2.3 Quality assessment

The quality of the included articles was assessed individually using JBI's critical appraisal tools.^16^ JBI's critical appraisal tools was chosen, as it has the broadest range of checklists for different study designs^17^ and therefore can handle that no restriction was made for the study design. The specific checklist used to assess each included article was chosen in accordance with the study design reported in the article. If the study design was not reported, the authors assessed and determined the design. Each included article was given a score based on the scoring system reported by Melo et al.^18^ A score of ≥70% was considered as a low risk of bias, a score between 50% and 69% as a moderate risk of bias, and a score of <49% as a high risk of bias.^18^

### 2.4 Data extraction and data synthesis

The information extracted from each included article was the trial design, sample size, demographics, clinical information, i.e., glycated hemoglobin (HbA1c), body mass index (BMI), and duration of T2D, treatment regimen, the adherence level to newer second‐line OAD, and the adherence quantification measure used. The type of adherence quantification measure was not restricted, as both the adherence level within and between the drug classes was grouped by the adherence quantification measure for comparison. In studies reporting the adherence to different types of OADs, the adherence quantification measure of a subgroup was only considered if the studied treatment was metformin and an OAD, marketed after 2012. The extracted information is presented in tables and the adherence to newer second‐line OADs are visualized in figures. The overall findings of this systematic review are summarized in a narrative synthesis.

### 2.5 Nomenclature of targets and ligands

Key protein targets and ligands in this article are hyperlinked to corresponding entries in http://www.guidetopharmacology.org, the common portal for data from the IUPHAR/BPS Guide to PHARMACOLOGY,^19^ and are permanently archived in the Concise Guide to PHARMACOLOGY 2019/20.^20^

## 3 RESULTS

**FIGURE 1:**
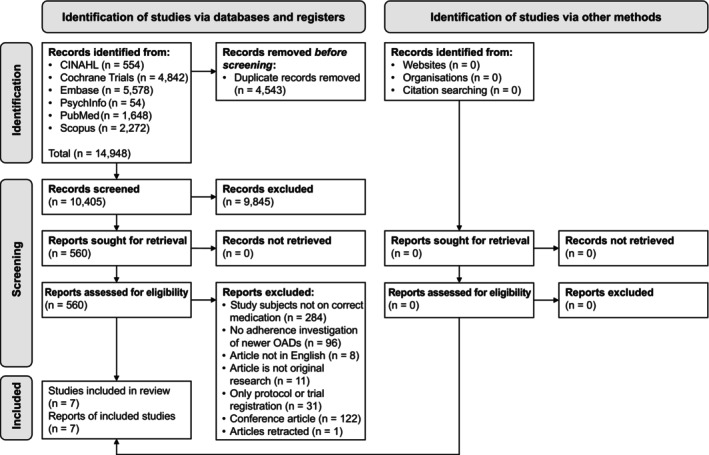
PRISMA 2020 flow diagram^14^ of the study selection process.

A total of 14 948 articles were identified through the systematic literature search. Seven articles met the criteria and were thus included as the final sample. The study selection process is reported in the PRISMA 2020 flow diagram shown in Figure 1.

### 3.1 Study characteristics

The characteristics of the included articles and study populations of these is reported in Table 1. All seven included studies were retrospective cohort studies using data from European countries (Hungary, Italy, Spain, and the United Kingdom) to determine the adherence level. The second‐line OADs investigated in these studies were the DPP4i alogliptin and the SGLT2is canagliflozin, dapagliflozin, and empagliflozin. Furthermore, Gordon et al.,^21^ Romagnoli et al.,^22^ and Strain et al.^23^ investigated adherence to SGLT2is, but did not report the type of SGLT2is being investigated.

**TABLE 1:** Overview of the included articles and the characteristics of the study population.

Author, Year	Country	Study type	Data source	Duration and time of trial	Second‐line OAD	Sample size	Age	Sex	T2D Duration	HbA1c
Gordon et al.^21^	UK	Cohort study	The Clinical Practice Research Datalink (CPRD) database.	Duration: Up to 365 days Time: 2008–2016	SGLT2i (unspecified)	232	Not reported	Not reported	Not reported	Not reported
Jermendy et al.^24^	Hungary	Cohort study	The National Institute of Health Insurance Fund Management database.	Duration: Up to 2 years Time: 2014–2017	SGLT2i (dapagliflozin or empagliflozin)	2603	Mean: 59.5 years SD: 9.4 years	Males: 50.5%	Not reported	Not reported
Rea et al.^25^	Lombardy, Italy	Cohort study	The Healthcare Utilization databases of Lombardy.	Duration: 1 year Time: 2007–2015	SGLT2i (canagliflozin, dapagliflozin or empagliflozin)	1276	69.5% was 40–64 years and 30.5% was ≥65 years.	Males: 62.3%	Not reported	Not reported
Romagnoli et al.^22^	Pescara, Italy	Cohort study	The administrative database of the pharmacy of the Hospital of Pescara, Italy.	Duration: Up to 3 years Time: 2011–2019	SGLT2i (unspecified)	757	Median: 65 years Min‐Max: 33–90 years	Males: 60%	Not reported	Not reported
Strain et al.^23^	UK	Cohort study	The Clinical Practice Research Datalink (CPRD) database.	Duration: Up to 2 years Time: 2012–2016	SGLT2i (unspecified)	490	Mean: 56.25 years SD: 9.80 years	Males: 56.1%	Mean: 4.21 years SD: 3.01 years	Mean: 8.38% SD: 0.80%
Vlacho et al.^26^	Catalonia, Spain	Cohort study	The SIDIAP (Information System for the Development of Research in Primary Care) database.	Duration: Up to 24 months Time: 2010–2017	DPP4i (alogliptin) SGLT2i (canagliflozin, dapagliflozin or empagliflozin)	Alogliptin: 22 Dapagliflozin: 539 Empagliflozin: 501 Canagliflozin: 175	Alogliptin: Not reported. SGLT2is: Mean: 60.5 years SD: 11.1 years	Alogliptin: Not reported. SGLT2is: Males: 56.5%	Alogliptin: Not reported SGLT2is: Mean: 7.89 years SD: 6.67 years	Alogliptin: Not reported SGLT2is: Mean: 8.77% SD: 1.49%
Wilding et al.^27^	UK	Cohort study	Clinical Practice Research Datalink (CPRD) database.	Duration: Up to 18 months Time: 2013–2016	SGLT2i (canagliflozin, dapagliflozin or empagliflozin)	441	Mean: 55.05 years SD: 10.14 years	Males: 60.3%	Median: 3.30 years IQR: 1.47–5.55	Mean: 9.07% SD: 1.58%

### 3.2 Quality assessment

As all included studies were cohort studies, and the quality assessment was based on JBI's critical appraisal checklist for cohort studies. The quality assessment of each included article is reported in Table 2.

**TABLE 2:** Quality assessment of the included articles based on JBI's critical appraisal tools.^16^

Article	JBI's critical appraisal checklist questions for cohort studies											Score	Risk of bias
	1	2	3	4	5	6	7	8	9	10	11		
Gordon et al.^21^	+	+	+	+	+	NA	+	+	+	+	+	10/10 100%	Low
Jermendy et al.^24^	U	+	+	+	−	NA	+	+	−	−	+	3/10 30%	High
Rea et al.^25^	+	+	+	+	+	NA	+	+	−	U	+	8/10 80%	Low
Romagnoli et al.^22^	+	+	+	U	−	NA	+	+	−	+	+	5/10 50%	Moderate
Strain et al.^23^	+	+	+	+	+	NA	+	+	−	−	+	6/10 60%	Moderate
Vlacho et al.^26^	+	+	+	+	+	NA	+	+	+	U	+	9/10 90%	Low
Wilding et al.^27^	+	+	+	+	+	NA	+	+	U	−	+	7/10 70%	Low

*Note*: For each question of the appraisal checklist for cohort studies, the answer is reported, and the total score is used to assess the risk of bias. A score of ≥70% was considered as a low risk of bias, a score between 50%–69% as moderate risk of bias, and a score of <49% as high risk of bias, as reported by Melo et al.^18^

Abbreviations: +: yes; −: no; NA, not applicable; U, unclear.

All but one of the included articles were evaluated to have a low to moderate risk of bias based on JBI's critical appraisal tools for cohort studies (Table 2). In contrast, the risk of bias in the article by Jermendy et al.^24^ was assessed to be high. The assessment of a high risk of bias in the article of Jermendy et al.^24^ is a result of that (1) it is unclear whether the study groups have similar characteristics, (2) no strategies were used to deal with confounding factors, and (3) the reasons of loss to follow‐up were not explored and methods to consider incomplete follow‐up was not used in the analysis.

### 3.3 Adherence to second‐line oral antidiabetic drugs

#### 3.3.1 Measurement methods

In the included articles, the objective measures to quantify adherence was (1) treatment discontinuation, (2) medication possession ratio (MPR), and (3) the ratio between the received daily dose (RDD) and prescribed daily dose (PDD). The reported measurement methods to ascertain adherence to second‐line OADs in the included articles are shown in Table 3.

**TABLE 3:** Overview of the measurement methods used to ascertain the adherence to second‐line OADs in the included articles.

Article	Measurement method
Gordon et al.^21^	MPR
Jermendy et al.^24^	Discontinued^a^ subjects at 12 months
Rea et al.^25^	Discontinued^b^ subjects at 12 months
Romagnoli et al.^22^	RDD/PDD
Strain et al.^23^	MPR + Discontinued^c^ subjects at 6 and 12 months
Vlacho et al.^26^	MPR + Discontinued^a^ subjects at 6, 12, and 24 months
Wilding et al.^27^	Discontinued^d^ subjects at 6, 12, and 18 months

*Note*: Discontinuation was defined as a period without treatment of a: ≥90 days, b: ≥60 days, c: not reported, and d: ≥184 days.

Abbreviations: MPR, medication possession ratio; PDD, prescribed daily dose; RDD, received daily dose.

A total of five articles^23^, ^24^, ^25^, ^26^, ^27^ reported the percentage of subjects discontinuing treatment with a second‐line OAD at given time points after treatment initiation. However, the duration of the treatment‐free period used to define treatment discontinuation, varied across the studies, spanning from 60 to 184 days^23^, ^24^, ^25^, ^26^, ^27^ (Table 3). Treatment discontinuation was defined as a change in the type of OAD by Jermendy et al.,^24^ Rea et al.,^25^ Strain et al.,^23^ and Vlacho et al.,^26^ whereas Wilding et al.^27^ also included treatment intensification as treatment discontinuation.

MPR is defined as the ratio between the number of days a patient is supplied with the given medication and the number of days between each medication refill over a given time span.^28^ It expresses the percentage of time that a subject has the medication available^28^ and is, thus, a proxy for treatment adherence. The commonly used definition of treatment adherence, in terms of MPR, is MPR ≥80%.^28^ The percentage of adherent subjects, in terms of MPR, was reported in three of the articles.^21^, ^23^, ^26^

#### 3.3.2 Treatment discontinuation

**FIGURE 2:**
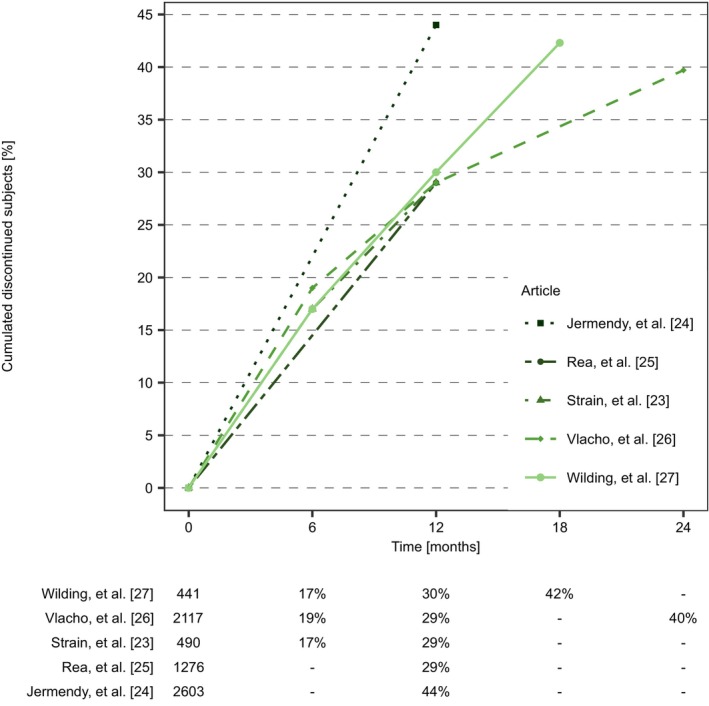
Cumulated discontinuation of SGLT2i as second‐line treatment among people with T2D on background metformin. The table shows the number of subjects initiating the treatment and the cumulated percentage of discontinued subjects at 6, 12, 18, and 24 months after treatment initiation. The hyphen (−) indicates that no discontinuation percentage was reported in the article of the study at the given time.

The five studies^23^, ^24^, ^25^, ^26^, ^27^ reporting treatment discontinuation solely focused on SGLT2is but did not report the type of SGLT2i. Thus, a comparison between the different SGLT2i types in terms of discontinuation was not possible. The percentage of subjects discontinuing SGLT2is as a second‐line treatment within the first 24 months after treatment initiation are shown in Figure 2.

The percentage of discontinued subjects 12 months after treatment initiation was reported by all five studies.^23^, ^24^, ^25^, ^26^, ^27^ Similar percentages of discontinued subjects at 12 months, approximately 30%, were reported by all except Jermendy et al.^24^ that reported a higher percentage of treatment discontinuation of 44% (Figure 2). Strain et al.^23^ and Wilding et al.^27^ reported treatment discontinuation based on data from the same database.

The course of the cumulated percentage of subjects discontinuing treatment are relatively similar across the five studies with a tendency of decreasing percentages of subjects discontinuing the treatment. A statistical test was not performed to compare the slopes, as a such test would have insufficient power due to the low number of samples.

#### 3.3.3 Medication possession ratio

**FIGURE 3:**
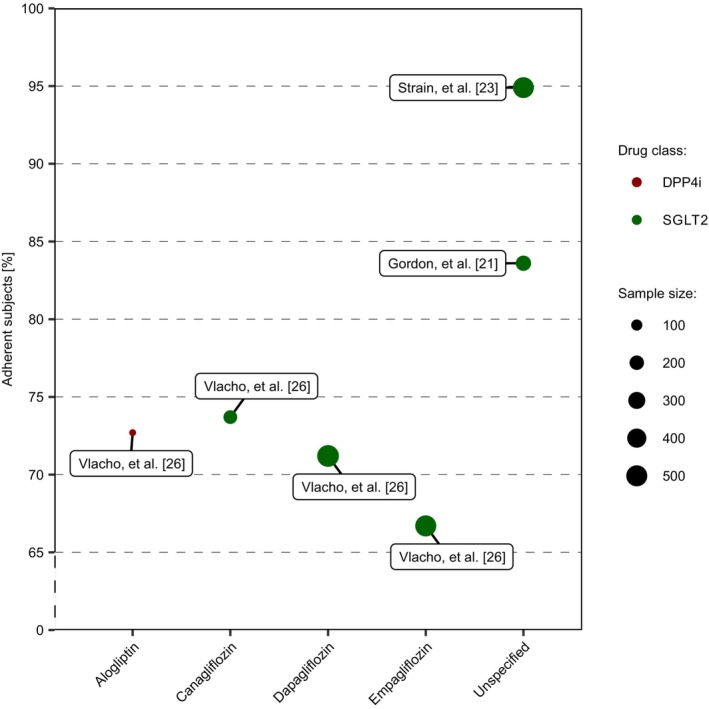
The percentage of adherent subjects grouped by drug class (DPP4i or SGLT2i) and type of drug. Adherence was defined as an MPR ≥80%. The size of each point represents the number of subjects in treatment with the given second‐line OAD. MPR, medication possession ratio; DPP4i, dipeptidyl peptidase 4 inhibitor; SGLT2i, sodium‐glucose cotransporter 2 inhibitor.

The percentage of adherent subjects, defined as subjects with an MPR ≥80%, by type and class of drug are shown in Figure 3. The reported percentage of adherent subjects ranged from 61.7% to 94.9%. The lowest percentage of adherent subjects was reported in a group treated with empagliflozin^26^ and the highest percentage was reported in subjects treated with an unspecified SGLT2i.^23^

The DPP4i alogliptin and the SGLT2is canagliflozin, dapagliflozin, and empagliflozin were compared by Vlacho et al.^26^ The percentage of subjects, which were adherent to alogliptin (72.7%) was comparable to that of canagliflozin (73.7%) and dapagliflozin (71.7%).^26^ However, the percentage of subjects, adherent to alogliptin, was based on 22 subjects.^26^ Among the three types of SGLT2i, the highest percentage of adherent subjects was reported in the group treated with canagliflozin (73.7%), followed by dapagliflozin (71.7%), and the lowest percentage of adherent subjects for empagliflozin (61.7%).^26^ The two studies investigating the unspecified types of SGLT2i both reported considerably higher percentages of SGLT2i adherent subjects (94.9% and 83.6%, respectively).

## 4 DISCUSSION

In this systematic review, the adherence to newer second‐line OADs among people with T2D on metformin was investigated. The reported adherence, defined as the percentage of subjects with MPR ≥80%, ranged between 61.7% and 94.9% across alogliptin, canagliflozin, dapagliflozin, empagliflozin, and unspecified types of SGLT2is in the included articles. Furthermore, the reported percentages of subjects discontinuing SGLT2is within the first 12 months after initiation ranged from 29% to 44%. The slope of the cumulated percentages of subjects discontinuing SGLT2is, reported across the included articles, had a decreasing tendency over the first 24 months after initiation, which could indicate that the risk of discontinuation is greatest shortly after initiation.

The percentage of adherent subjects to OADs, defined as an MPR ≥80%, have previously been investigated in systematic reviews and meta‐analyses.^7^, ^8^ In the meta‐analysis by Iglay et al.^8^ from 2015, the percentage of adherent subjects was reported across nine studies, which analyzed metformin, sulfonylureas, TZDs, alpha‐glucosidase inhibitors, meglitinides, DPP4is, and unspecified types of OADs. Iglay et al.^8^ found that 48% to 89% of the subjects across the studies and different OAD types were adherent. Similar results were found by Krass et al.^7^ in their systematic review from 2014. The percentage of adherent subjects was 46%–89% across four studies, which investigated adherence to metformin, pioglitazone, sulfonylurea, and unspecified types of OADs. The results reported in these previous studies showed that the percentage range of adherent subjects to newer second‐line OADs, investigated in the present systematic review, was within the upper part of the ranges reported by Krass et al.^7^ and Iglay et al.^8^ This suggests that the adherence to newer second‐line OADs might be greater than that to older OADs. A firm conclusion can, nonetheless, not be drawn as no direct comparison between the MPR of newer and older OADs was conducted.

The percentage of subjects, which remained persistent to OADs 12 months after intervention, has been investigated in two previously published systematic reviews. In the systematic review by Cramer^6^ from 2004, 16%–58% of the subjects persisted on the prescribed OADs for at least 12 months after initiation across five studies, which investigated acarbose, glipizide, and unspecified types of OADs. Odegard and Capoccia^29^ reported similar results in their systematic review from 2007, in which 16%–44% of subjects persisted a minimum of 12 months after treatment initiation. The 12‐month persistence reported by Odegard and Capoccia^29^ was based on two studies, which investigated acarbose and glipizide. This indicates that the persistence of newer second‐line OADs seems to be better compared to that of older OADs, as the 12‐month discontinuation for the newer second‐line OADs investigated in the present systematic review was 29%–44%. However, as no direct comparison were made between the newer and older second‐line OADs, a final conclusion cannot be drawn.

In this systematic review, the OAD with the highest percentage of adherent subjects in terms of MPR was canagliflozin when compared to that of alogliptin, dapagliflozin, and empagliflozin. These four OADs differ in attributes such as associated side effects and dosing requirements, which may influence the adherence. The patients' preferences regarding the attributes of newer OADs, were investigated by Savarese et al.^30^ in a survey of 553 people with T2D from the United States. In the survey, the patients ranked risk of genital infections highest, followed by reduction of body weight, dosing conditions involving fasting and food restrictions, risk of vomiting, and risk reduction of cardio vascular death.^30^ SGLT2is i.e., canagliflozin, dapagliflozin, and empagliflozin are associated with an increased risk of genital infection,^31^, ^32^ whereas DPP4is, i.e., alogliptin are not.^32^ The SGLT2is are, nonetheless, associated with weight loss^32^ and reduced risk of cardiovascular complications^31^ while the DPP4is might slightly increase body weight^32^ and have no effect on cardiovascular complications.^31^ Neither DPP4is nor SGLT2is have an increased risk of gastrointestinal side effects.^32^ The method of administration differs in complexity, also within the class of SGLT2i. Alogliptin, dapagliflozin and empagliflozin have the least complex method of administration, as these can be taken with or without food,^12^, ^33^, ^34^ whereas canagliflozin should be administered prior to the first meal of the day.^35^ Despite that canagliflozin, as an SGLT2i, is associated with an increased risk of genital infection, which according to the survey by Savarese et al.^30^ is ranked as the biggest consideration, and has the most complex administration method of the three SGLT2is, it had the highest reported percentage of adherent patients. However, these adherence data was all reported by Vlacho et al.^26^ and the sample sizes were relatively small. Thus, more data is needed to investigate the influence of potential adherence barriers on treatment adherence.

The adherence was quantified based on data from pharmacy claims and registries in the included articles. As it is not possible to verify whether a subject actually takes the prescribed medication, the prescribed dosage, and abides by the prescribed method of administration, the adherence might be overestimated. Furthermore, overlapping medication supply is not taken into consideration in the calculation of MPR, which can also result in an overestimation. However, adherence quantification measures based on data from pharmacy claims and registries can be used as proxies for adherence and are commonly used methods to quantify adherence.^28^

In the included studies, the most reported adherence quantification measures were MPR and treatment discontinuation. Thus, it was possible to investigate two distinct aspects of treatment adherence, namely how long the subjects remained on the treatment and to what degree the subjects took the medication while on the treatment. However, both adherence quantification measures were defined differently across the included articles. The reported treatment discontinuation varied across the studies in terms of when a subject was considered as discontinued on the treatment and how loss to follow‐up was managed. Similarly, the reported MPR was calculated over different durations across the studies and differed in terms of whether loss to follow‐up was considered in the calculations. Thus, the reported adherence quantification measures may not be directly comparable across the studies but provides an indication for the adherence level.

This systematic review is based on a systematic literature search, which was conducted in a highly systematic way in seven different databases to ensure all relevant articles were retrieved. The design of the systematic literature search was made with input from research librarians at Aalborg University Library and at the Medical Library of Aalborg University Hospital to ensure a high quality of the literature search.

The main limitation of this systematic review is the relatively few included studies. Thus, no statistical tests were performed, as power would be too low due to the low number of studies. Instead, the conclusions have been moderated to reflect the low number of included studies. Additionally, all the included studies were conducted in European countries, which may introduce a bias, as the diversity of the study population is limited and might not be representative outside of Europe.

## 5 CONCLUSIONS

The reported findings of SGLT2i discontinuation over time from treatment initiation had a decreasing tendency that might indicate that the treatment discontinuation risk is greatest immediately after initiation. Across the included studies, between 29% and 44% of subjects on SGLT2i discontinued their treatment within the first 12 months of treatment initiation. In terms of MPR, 61.7%–94.9% of subjects treated with either alogliptin, canagliflozin, dapagliflozin, empagliflozin or an unspecified type of SGLT2i were adherent. Overall, both adherence quantification measures indicate that the adherence to the newer second‐line OADs may be better than that of older OADs but should be verified with a study directly comparing older and newer OADs.

## AUTHOR CONTRIBUTIONS

All authors contributed to the design of the protocol of this systematic review and provided input to the final version of the manuscript. NSHC performed the systematic literature search and data extraction with guidance from authors CD, MHJ, and SH. The data analysis and interpretations were made by NSHC with substantial contributions from CD and MHJ. All authors have read and approved the final version of the manuscript.

## FUNDING INFORMATION

This work was supported by a research grant from the Danish Diabetes and Endocrine Academy, which is funded by the Novo Nordisk Foundation, grant number NNF17SA0031406.

## CONFLICT OF INTEREST STATEMENT

The authors Dethlefsen, Holdt‐Caspersen, and Jensen are employees of Novo Nordisk and hold shares herein. Peter Vestergaard is the Head of Research at Steno Diabetes Center North Denmark funded by the Novo Nordisk Foundation.

## ETHICS STATEMENT

Approval from an ethics committee was not necessary, as this was not a clinical trial.

## Supporting information


Data S1.

## Data Availability

Data can be made available upon request from the corresponding author.
