# Supplementary material for: Adherence to newer second‐line oral antidiabetic drugs among people with type 2 diabetes—A systematic review
Source: Pharmacol Res Perspect. 2024 Mar 7;12(2):e1185. doi: 10.1002/prp2.1185 (PMC10918987; doi:10.1002/prp2.1185)
Supplement: Supplementary file 1 — Data S1. [file PRP2-12-e1185-s001.docx]

Supplementary File

Table of Contents

[1 Protocol of the Systematic Literature Search 2](#_Toc147758478)

[1.1 Research Question 2](#_Toc147758479)

[1.2 Databases 2](#_Toc147758480)

[1.3 Search Strategy 2](#_Toc147758481)

[1.4 Inclusion and Exclusion Criteria 5](#_Toc147758482)

[1.4.1 Inclusion Criteria 5](#_Toc147758483)

[1.4.2 Exclusion Criteria 5](#_Toc147758484)

[1.5 Screening of Articles 5](#_Toc147758485)

[1.6 Evaluation of Articles 6](#_Toc147758486)

[1.7 Information Extraction from Included Articles 6](#_Toc147758487)

[1.8 Documentation 6](#_Toc147758488)

[2 Search String and Retrieved Articles for each Database 6](#_Toc147758489)

[2.1 Embase 6](#_Toc147758490)

[2.2 PubMed 8](#_Toc147758491)

[2.3 CINAHL 11](#_Toc147758492)

[2.4 PsycInfo 15](#_Toc147758493)

[2.5 Cochrane Trials 18](#_Toc147758494)

[2.6 Scopus 20](#_Toc147758495)

# Protocol of the Systematic Literature Search

## Research Question

What is the level of adherence to oral second line antidiabetic medication amongst people with type 2 diabetes?

## Databases

The literature search will be performed in the following databases:

- APA PsychInfo.
- CINAHL (EBSCO)
- Cochrane
- Embase.com
- PubMed.
- Scopus

## Search Strategy

The systematic literature search will be performed as a block search with the search blocks ‘*Type 2 diabetes’*, ‘*Second line oral antidiabetics’,* and ‘*Adherence’*. Each block consists of relevant synonyms (both as free text and a thesaurus term if one exists). The thesaurus search terms will be specified to each database. The generic search terms of each search block of the systematic literature search are shown in Tabel 1. The search will be restricted to include articles published from 2012 and forwards, as systematic reviews assessing the adherence to antidiabetic medications exist.

Tabel 1: Generic search terms for each search block.

| ***Type 2 diabetes*** | **A**  **N**  **D** | ***Second-line  oral antidiabetics*** | | | **A**  **N**  **D** | ***Adherence*** |
| --- | --- | --- | --- | --- | --- | --- |
| *"Diabetes Mellitus, Type 2"[Mesh]*  *“Type 2 diabetes”[Text Word]*  *“Type 2 diabetes mellitus”[Text Word]*  *“Type ii diabetes”[Text Word]*  *“Type ii diabetes mellitus”[Text Word]*  *T2D[Text Word]*  *T2DM[Text Word]*  *“non-insulin dependent diabetes”[Text Word]*  *NNIDM[Text Word]*  *“Diabetes type 2”[Text Word]*  *“Diabetes type ii”[Text Word]*  *“Diabetes mellitus type 2”[Text Word]*  *“Diabetes mellitus type ii”[Text Word]*  *PWT2D[Text Word]* |  | *OHA[Text Word]*  *OGLA[Text Word]*  *OAD[Text Word]*  *"Dipeptidyl-Peptidase IV Inhibitor*"[Mesh]*  *“DPP-4 inhibitor*”[Text Word]*  *“DPP 4 inhibitor*”[Text Word]*  *“DPP-IV inhibitor*”[Text Word]*  *“DPP IV inhibitor*”[Text Word]*  *“DPP4 inhibitor*”[Text Word]*  *gliptin*[Text Word]*  *“Dipeptidyl Peptidase IV Inhibitor*”[Text Word]*  *“Dipeptidyl Peptidase 4 Inhibitor*”[Text Word]*  *“Dipeptidyl-Peptidase IV Inhibitor*”[Text Word]*  *“Dipeptidyl-Peptidase 4 Inhibitor*”[Text Word]*  *“Dipeptidyl Peptidase-IV Inhibitor*”[Text Word]*  *“Dipeptidyl Peptidase-4 Inhibitor*”[Text Word]*  *“Dipeptidyl-Peptidase-IV Inhibitor*”[Text Word]*  *“Dipeptidyl-Peptidase-4 Inhibitor*”[Text Word]*  *alogliptin[Supplementary Concept]*  *alogliptin[Text Word]*  *Linagliptin[Mesh]*  *Linagliptin[Text Word]*  *saxagliptin [Supplementary Concept]*  *saxagliptin*[Text Word]*  *"Sitagliptin Phosphate"[Mesh]*  *Sitagliptin*[Text Word]*  *"Sulfonylurea Compounds"[Mesh]*  *Sulfonylurea*[Text Word]*  *SU[Text Word]*  *Gliclazid*[Text Word]*  *Glimepirid*[Text Word]*  *"Sodium-Glucose Transporter 2 Inhibitors"[MeSH Terms]*  *"Sodium-Glucose Transporter 2 Inhibitor*"[Text Word]*  *"Sodium-Glucose Transporter-2 Inhibitor*"[Text Word]*  *“Sodium-glucose co-transporter-2 inhibitor*”[Text Word]*  *“Sodium-glucose co-transporter 2 inhibitor*”[Text Word]*  *"Sodium Glucose Transporter-2 Inhibitor*"[Text Word]*  *"Sodium Glucose Transporter 2 Inhibitor*"[Text Word]*  *“Sodium glucose co-transporter-2 inhibitor*”[Text Word]*  *“Sodium glucose co-transporter 2 inhibitor*”[Text Word]*  *“SGLT-2 inhibitor*”[Text Word]*  *“SGLT2 inhibitor*”[Text Word]*  *“SGLT 2 inhibitor*”[Text Word]*  *Gliflozin*[Text Word]*  *Canagliflozin[Mesh]*  *Canagliflozin*[Text Word]*  *dapagliflozin[Supplementary Concept]*  *dapagliflozin*[Text Word]*  *empagliflozin[Supplementary Concept]*  *empagliflozin*[Text Word]* | | |  | *"Medication Adherence"[Mesh]*  *adhere*[Text Word]*  *nonadhere*[Text Word]*  *complian*[Text Word]*  *noncomplian*[Text Word]*  *comply*[Text Word]*  *noncomply*[Text Word]*  *persist*[Text Word]*  *nonpersist*[Text Word]*  *discontin*[Text Word]*  *concord*[Text Word]*  *omit*[Text Word]* |
|  |  | **OR** | | |  |  |
|  |  | *Oral[Text Word]*  *Orally[Text Word]*  *"Administration, Oral"[Mesh:NoExp]* | **A**  **N**  **D** | *"Hypoglycemic Agents"[Mesh]*  *hypoglycemic*[Text Word]*  *hypoglycaemic*[Text Word]*  *antidiabetic*[Text Word]*  *antihyperglycaemic*[Text Word]*  *antihyperglycemic*[Text Word]*  *“glucose-lowering”[Text Word]*  *“glucagon-like peptide 1 receptor agonist*”[Text Word]*  *“glucagon-like peptide-1 receptor agonist*”[Text Word]*  *"GLP-1 receptor agonist*”[Text Word]*  *"GLP-1-RA*”[Text Word]*  *"GLP-1RA*”[Text Word]* |  |  |

## Inclusion and Exclusion Criteria

### Inclusion Criteria

1. The study subjects are diagnosed with type 2 diabetes.
2. The study group is on treatment with metformin and an oral second line antidiabetics (i.e., oral GLP-1 RA, DDP4-inhibitors, SGLT-2 inhibitors, and SU) marketed from 2012 and forwards.
3. The study investigates adherence to treatment with oral second line antidiabetics (i.e., oral GLP-1 RA, DDP4-inhibitors, SGLT-2 inhibitors, and SU) marketed from 2012 and forwards.
4. The article must be available in full text.
5. The article must be written in English.
6. The article must be original research.

### Exclusion Criteria

1. The study subjects are younger than 18 years old.
2. The study subjects are diagnosed with diabetes other than type 2.
3. The study has an inclusion that subjects have comorbidities
4. The study was set during special circumstances, such as Ramadan or the COVID-19 pandemic.
5. Published prior to 2012.

## Screening of Articles

The articles, retrieved from each of the five databases, are pooled and duplicates removed. The remaining articles are screened for relevance cf. the inclusion and exclusion criteria in three steps: 1) based on title, 2) based on abstract, and 3) based on full text. Articles, which either cite or are cited by one or more of the included articles, are screened as described above and included if the criteria are fulfilled.

## Evaluation of Articles

All articles are evaluated using the Joanna Briggs Institutes’ critical appraisal tools.

## Information Extraction from Included Articles

The following information will be retrieved from the included articles:

- Trial design:
  - Short description of the trial design.
  - Control group?
  - Blinding?
  - Trial duration.
  - Statistical considerations (sample size, statistical power, etc.)
- Subjects:
  - Number (control and intervention).
  - Demographics (age, sex, country, ethnicity, T2D duration).
  - Treatment regimen.
  - HbA1c.
- Adherence:
  - How is adherence measured/evaluated?
  - What is the reported adherence?
  - If investigated, which factors influenced adherence and how?

## Documentation

The retrieval of articles will be documented for each database in terms of used search terms (free text, including wildcards, and thesaurus terms), combination of search terms into search blocks, number of articles retrieved, and the date at which the search was performed.

The screening process will be illustrated as a PRISMA flowchart. The information retrieved from the included articles is summarised and presented as a review.

# Search String and Retrieved Articles for each Database

## Embase

| Search | Search String | Results |
| --- | --- | --- |
| **#1 (T2D)** | 'non insulin dependent diabetes mellitus'/exp OR  'type 2 diabetes' OR  'type 2 diabetes mellitus':ab,ti OR  'type ii diabetes':ab,ti OR  'type ii diabetes mellitus':ab,ti OR  't2d':ab,ti OR 't2dm':ab,ti OR  'non-insulin dependent diabetes':ab,ti OR  'niddm':ab,ti OR  'diabetes type 2':ab,ti OR  'diabetes type ii':ab,ti OR  'diabetes mellitus type 2':ab,ti OR  'diabetes mellitus type ii':ab,ti OR  'pwt2d':ab,ti | 361,275 |
| **#2 (OAD 1)** | 'oral':ab,ti OR  'orally':ab,ti OR  'oral drug administration'/exp | 1,326,945 |
| **#3 (OAD 2)** | 'antidiabetic agent'/de OR  'hypoglycemic*':ab,ti OR  'hypoglycaemic*':ab,ti OR  'antidiabetic*':ab,ti OR  'antihyperglycaemic*':ab,ti OR  'antihyperglycemic*':ab,ti OR  'glucose-lowering':ab,ti OR  'glucagon like peptide 1 receptor agonist*':ab,ti OR  'glp 1 receptor agonist*':ab,ti OR  'glp 1 ra*':ab,ti OR  'glp 1ra*':ab,ti OR  'glucagon like peptide 1 receptor agonist'/de | 133,363 |
| **#4 (OAD 3)** | #2 AND #3 | 31,847 |
| **#5 (OAD 4)** | 'oha':ab,ti OR  'ogla':ab,ti OR  'oad':ab,ti OR  'oral antidiabetic agent'/exp OR  'dipeptidyl peptidase iv inhibitor'/de OR  'dpp 4 inhibitor*':ab,ti OR  'dpp iv inhibitor*':ab,ti OR  'dpp4 inhibitor*':ab,ti OR  'gliptin*':ab,ti OR  'dipeptidyl peptidase iv inhibitor*':ab,ti OR  'dipeptidyl peptidase 4 inhibitor*':ab,ti OR  'alogliptin':ab,ti OR  'sulfonylurea derivative'/exp OR  'sulfonylurea*':ab,ti OR  'su':ab,ti OR  'sodium glucose cotransporter 2 inhibitor'/exp OR  'sodium glucose transporter 2 inhibitor*':ab,ti OR  'sodium glucose co transporter 2 inhibitor*':ab,ti OR  'sodium glucose cotransporter 2 inhibitor*':ab,ti OR  'sglt 2 inhibitor*':ab,ti OR  'sglt2 inhibitor*':ab,ti OR  'gliflozin*':ab,ti OR  'canagliflozin*':ab,ti OR  'dapagliflozin*':ab,ti OR  'empagliflozin*':ab,ti OR  'ertugliflozin*':ab,ti OR  'thiazolidinedione*':ab,ti OR  'tzd*':ab,ti OR  '2,4 thiazolidinedione derivative'/exp OR  'glitazone*':ab,ti | 214,629 |
| **#6 (OAD Total)** | #4 OR #5 | 226,931 |
| **#7 (Adherence)** | 'adherence'/exp OR  'patient compliance'/exp OR  'concordance'/exp OR  'adhere*':ab,ti OR  'non-adhere*':ab,ti OR  'nonadhere*':ab,ti OR  'complian*':ab,ti OR  'noncomplian*':ab,ti OR  'non-complian*':ab,ti OR  'comply*':ab,ti OR  'noncomply*':ab,ti OR  'non-comply*':ab,ti OR  'persist*':ab,ti OR  'nonpersist*':ab,ti OR  'non-persist*':ab,ti OR  'discontin*':ab,ti OR  'concord*':ab,ti OR  'omit*':ab,ti | 1,689,269 |
| **#8 (Combined)** | #1 AND #6 AND #7 | 8,330 |
| **Date and language restrictions** | #8 AND [2012-2022]/py AND [english]/lim | 5,578 |
| Date of Search | 12-JUL-2022 |  |

## PubMed

| Search | Search String | Results |
| --- | --- | --- |
| **#1 (T2D)** | "diabetes mellitus, type 2"[MeSH Terms] OR  "Type 2 diabetes"[Text Word] OR  "Type 2 diabetes mellitus"[Text Word] OR  "Type ii diabetes"[Text Word] OR  "Type ii diabetes mellitus"[Text Word] OR  "T2D"[Text Word] OR  "T2DM"[Text Word] OR  "non-insulin dependent diabetes"[Text Word] OR  "NIDDM"[Text Word] OR  "Diabetes type 2"[Text Word] OR  "Diabetes type ii"[Text Word] OR  "Diabetes mellitus type 2"[Text Word] OR  "Diabetes mellitus type ii"[Text Word] OR  "PWT2D"[Text Word] | 223,012 |
| **#2 (OAD 1)** | "Oral"[Text Word] OR  "Orally"[Text Word] OR  "administration, oral"[MeSH Terms:noexp] | 833,374 |
| **#3 (OAD 2)** | "Hypoglycemic Agents"[MeSH Terms] OR  "hypoglycemic*"[Text Word] OR  "hypoglycaemic*"[Text Word] OR  "antidiabetic*"[Text Word] OR  "antihyperglycaemic*"[Text Word] OR  "antihyperglycemic*"[Text Word] OR  "glucose-lowering"[Text Word] OR  "glucagon like peptide 1 receptor agonist*"[Text Word] OR  "glucagon like peptide 1 receptor agonist*"[Text Word] OR  "glp 1 receptor agonist*"[Text Word] OR  "glp 1 ra*"[Text Word] OR  "glp 1ra*"[Text Word] | 116,717 |
| **#4 (OAD 3)** | #2 AND #3 | 22,717 |
| **#5 (OAD 4)** | "OHA"[Text Word] OR  "OGLA"[Text Word] OR  "OAD"[Text Word] OR  "OHA"[Text Word] OR  "OGLA"[Text Word] OR  "OAD"[Text Word] OR  "dipeptidyl peptidase iv inhibitor*"[MeSH Terms] OR  "dpp 4 inhibitor*"[Text Word] OR  "dpp 4 inhibitor*"[Text Word] OR  "dpp iv inhibitor*"[Text Word] OR  "dpp iv inhibitor*"[Text Word] OR  "dpp4 inhibitor*"[Text Word] OR  "gliptin*"[Text Word] OR  "dipeptidyl peptidase iv inhibitor*"[Text Word] OR  "dipeptidyl peptidase 4 inhibitor*"[Text Word] OR  "dipeptidyl peptidase iv inhibitor*"[Text Word] OR  "dipeptidyl peptidase 4 inhibitor*"[Text Word] OR  "dipeptidyl peptidase iv inhibitor*"[Text Word] OR  "dipeptidyl peptidase 4 inhibitor*"[Text Word] OR  "dipeptidyl peptidase iv inhibitor*"[Text Word] OR  "dipeptidyl peptidase 4 inhibitor*"[Text Word] OR  "alogliptin"[Supplementary Concept] OR  "alogliptin"[Text Word] OR  "Sulfonylurea Compounds"[MeSH Terms] OR  "sulfonylurea*"[Text Word] OR  "SU"[Text Word] OR  "Sodium-Glucose Transporter 2 Inhibitors"[MeSH Terms] OR  "sodium glucose transporter 2 inhibitor*"[Text Word] OR  "sodium glucose transporter 2 inhibitor*"[Text Word] OR  "sodium glucose co transporter 2 inhibitor*"[Text Word] OR  "sodium glucose co transporter 2 inhibitor*"[Text Word] OR  "sodium glucose transporter 2 inhibitor*"[Text Word] OR  "sodium glucose transporter 2 inhibitor*"[Text Word] OR  "sodium glucose co transporter 2 inhibitor*"[Text Word] OR  "sodium glucose co transporter 2 inhibitor*"[Text Word] OR  "sglt 2 inhibitor*"[Text Word] OR  "sglt2 inhibitor*"[Text Word] OR  "sglt 2 inhibitor*"[Text Word] OR  "gliflozin*"[Text Word] OR  "canagliflozin"[MeSH Terms] OR  "canagliflozin*"[Text Word] OR  "dapagliflozin"[Supplementary Concept] OR  "dapagliflozin*"[Text Word] OR  "empagliflozin"[Supplementary Concept] OR  "empagliflozin*"[Text Word] OR  "ertugliflozin" [Supplementary Concept] OR  "ertugliflozin*"[Text Word] OR  "Thiazolidinediones"[Mesh] OR  "Thiazolidinedione*"[Text Word] OR  "tzd"[Text Word] OR  "glitazone*"[Text Word] | 64,544 |
| **#6 (OAD Total)** | #4 OR #5 | 79,795 |
| **#7 (Adherence)** | "Medication Adherence"[MeSH Terms] OR  "adhere*"[Text Word] OR  "non-adhere*"[Text Word] OR  "complian*"[Text Word] OR  "noncomplian*"[Text Word] OR  "comply*"[Text Word] OR  "noncomply*"[Text Word] OR  "persist*"[Text Word] OR  "nonpersist*"[Text Word] OR  "discontin*"[Text Word] OR  "concord*"[Text Word] OR  "omit*"[Text Word] | 1,184,004 |
| **#8 (Combined)** | #1 AND #6 AND #7 | 2,661 |
| **Date and language restrictions** | #8 AND ((2012:2022[pdat]) AND (english[Filter])) | 1,648 |
| Date of Search | 12-JUL-2022 |  |

## CINAHL

| Search | Search String | Results |
| --- | --- | --- |
| **#1 (T2D)** | (MH "Diabetes Mellitus, Type 2") OR  (TI type 2 diabetes) OR  (AB type 2 diabetes) OR  (TI Type 2 diabetes mellitus) OR  (AB Type 2 diabetes mellitus) OR  (TI Type ii diabetes) OR  (AB Type ii diabetes) OR  (TI Type ii diabetes mellitus) OR  (AB Type ii diabetes mellitus) OR  (TI T2D) OR  (AB T2D) OR  (TI T2DM) OR  (AB T2DM) OR  (TI non-insulin dependent diabetes) OR  (AB non-insulin dependent diabetes) OR  (TI NIDDM) OR  (AB NIDDM) OR  (TI Diabetes type 2) OR  (AB Diabetes type 2) OR  (TI Diabetes type ii) OR  (AB Diabetes type ii) OR  (TI Diabetes mellitus type 2) OR  (AB Diabetes mellitus type 2) OR  (TI Diabetes mellitus type ii) OR  (AB Diabetes mellitus type ii) OR  (TI PWT2D) OR  (AB PWT2D) | 88,557 |
| **#2 (OAD 1)** | (TI Oral) OR  (AB Oral) OR  (TI Orally) OR  (AB Orally) OR  (MH "Administration, Oral") | 167,765 |
| **#3 (OAD 2)** | (MH "Hypoglycemic Agents") OR  TI hypoglycemic* OR  AB hypoglycemic* OR  TI hypoglycaemic* OR  AB hypoglycaemic* OR  TI antidiabetic* OR  AB antidiabetic* OR  TI antihyperglycaemic* OR  AB antihyperglycaemic* OR  TI antihyperglycemic* OR  AB antihyperglycemic* OR  TI glucose-lowering OR  AB glucose-lowering OR  TI glucagon like peptide 1 receptor agonist* OR  AB glucagon like peptide 1 receptor agonist* OR  TI glucagon like peptide 1 receptor agonist* OR  AB glucagon like peptide 1 receptor agonist* OR  TI glp 1 receptor agonist* OR  AB glp 1 receptor agonist* OR  TI glp 1 ra* OR AB glp 1 ra* OR  TI glp 1ra* OR AB glp 1ra* OR (MH "Glucagon-Like Peptide-1 Receptor Agonists") | 26,181 |
| **#4 (OAD 3)** | #2 AND #3 | 4,982 |
| **#5 (OAD 4)** | (TI OHA) OR  (AB OHA) OR  (TI OGLA) OR  (AB OGLA) OR  (TI OAD) OR  (AB OAD) OR  (MH "Dipeptidyl Peptidase 4 Inhibitors+") OR  (TI dpp 4 inhibitor*) OR  (AB dpp 4 inhibitor*) OR  (TI dpp 4 inhibitor*) OR  (AB dpp 4 inhibitor*) OR  (TI dpp iv inhibitor*) OR  (AB dpp iv inhibitor*) OR  (TI dpp iv inhibitor*) OR  (AB dpp iv inhibitor*) OR  (TI dpp4 inhibitor*) OR  (AB dpp4 inhibitor*) OR  (TI gliptin*) OR  (AB gliptin*) OR  (TI dipeptidyl peptidase iv inhibitor*) OR  (AB dipeptidyl peptidase iv inhibitor*) OR  (TI dipeptidyl peptidase 4 inhibitor*) OR  (dipeptidyl peptidase 4 inhibitor*) OR  (TI dipeptidyl peptidase iv inhibitor*) OR  (AB dipeptidyl peptidase iv inhibitor*) OR  (TI dipeptidyl peptidase 4 inhibitor*) OR  (AB dipeptidyl peptidase 4 inhibitor*) OR  (TI dipeptidyl peptidase iv inhibitor*) OR  (AB dipeptidyl peptidase iv inhibitor*) OR  (TI dipeptidyl peptidase 4 inhibitor*) OR  (AB dipeptidyl peptidase 4 inhibitor*) OR  (TI dipeptidyl peptidase iv inhibitor*) OR  (AB dipeptidyl peptidase iv inhibitor*) OR  (TI dipeptidyl peptidase 4 inhibitor*) OR  (AB dipeptidyl peptidase 4 inhibitor*) OR  (TI alogliptin) OR  (AB alogliptin) OR  (MH "Sulfonylurea Compounds+") OR  (TI sulfonylurea*) OR  (AB sulfonylurea*) OR  (TI SU) OR  (AB SU) OR  (MH "Sodium-Glucose Co-Transporter 2 Inhibitors+") OR  (TI sodium glucose transporter 2 inhibitor*) OR  (AB sodium glucose transporter 2 inhibitor*) OR  (TI sodium glucose co transporter 2 inhibitor*) OR  (AB sodium glucose co transporter 2 inhibitor*) OR  (TI sodium glucose cotransporter 2 inhibitor*) OR  (AB sodium glucose cotransporter 2 inhibitor*) OR  (TI sglt 2 inhibitor*) OR  (AB sglt 2 inhibitor*) OR  (TI sglt2 inhibitor*) OR  (AB sglt2 inhibitor*) OR  (TI gliflozin*) OR  (AB gliflozin*) OR  (TI canagliflozin*) OR  (AB canagliflozin*) OR  (TI dapagliflozin*) OR  (AB dapagliflozin*) OR  (TI ertugliflozin*) OR  (AB ertugliflozin*) OR  (MH "Thiazolidinediones") OR  (TI thiazolidinedione*) OR  (AB thiazolidinedione*) OR  (TI tzd*) OR  (AB tzd*) OR  (TI glitazone*) OR  (AB glitazone*) | 24,832 |
| **#6 (OAD Total)** | #4 OR #5 | 28,297 |
| **#7 (Adherence)** | (MH "Patient Compliance+") OR  (TI concordance) OR  (AB concordance) OR  (TI adhere*) OR  (AB adhere*) OR  (TI non-adhere*) OR  (AB non-adhere*) OR  (TI nonadhere*) OR  (AB nonadhere*) OR  (TI complian*) OR  (AB complian*) OR  (TI noncomplian*) OR  (AB noncomplian*) OR  (TI non-complian*) OR  (AB non-complian*) OR  (TI comply*) OR  (AB comply*) OR  (TI noncomply*) OR  (AB noncomply*) OR  (TI non-comply*) OR  (AB non-comply*) OR  (TI persist*) OR  (AB persist*) OR  (TI nonpersist*) OR  (AB nonpersist*) OR  (TI non-persist*) OR  (AB non-persist*) OR  (TI discontin*) OR  (AB discontin*) OR  (TI concord*) OR  (AB concord*) OR  (TI omit*) OR  (AB omit*) | 284,469 |
| **#8 (Combined)** | #1 AND #6 AND #7 | 934 |
| **Date and language restrictions** | #8 AND Limiters - Published Date: 20120101-20221231; English Language | 554 |
| Date of Search | 12-JUL-2022 |  |

## PsycInfo

| Search | Search String | Results |
| --- | --- | --- |
| **#1 (T2D)** | it: (Type 2 Diabetes) OR  ti: (Type 2 diabetes) OR  ab: (Type 2 diabetes) OR  ti: (Type 2 diabetes mellitus) OR  ab: (Type 2 diabetes mellitus) OR  ti: (Type ii diabetes) OR  ab: (Type ii diabetes) OR  ti: (Type ii diabetes mellitus) OR  ab: (Type ii diabetes mellitus) OR  ti: (T2D) OR  ab: (T2D) OR  ti: (T2DM) OR  ab: (T2DM) OR  ti: (non-insulin dependent diabetes) OR  ab: (non-insulin dependent diabetes) OR  ti: (NIDDM) OR  ab: (NIDDM) OR  ti: (Diabetes type 2) OR  ab: (Diabetes type 2) OR  ti: (Diabetes type ii) OR  ab: (Diabetes type ii) OR  ti: (Diabetes mellitus type 2) OR  ab: (Diabetes mellitus type 2) OR  ti: (Diabetes mellitus type ii) OR  ab: (Diabetes mellitus type ii) OR  ti: (PWT2D) OR  ab: (PWT2D) | 10,516 |
| **#2 (OAD 1)** | ti: (Oral) OR  ab: (Oral) OR  ti: (Orally) OR  ab: (Orally) | 49,385 |
| **#3 (OAD 2)** | ti: (hypoglycemic*) OR  ab: (hypoglycemic*) OR  ti: (hypoglycaemic*) OR  ab: (hypoglycaemic*) OR  ti: (antidiabetic*) OR  ab: (antidiabetic*) OR  ti: (antihyperglycaemic*) OR  ab: (antihyperglycaemic*) OR  ti: (antihyperglycemic*) OR  ab: (antihyperglycemic*) OR  ti: (glucose-lowering) OR  ab: (glucose-lowering) OR  ti: (glucagon like peptide 1 receptor agonist*) OR  ab: (glucagon like peptide 1 receptor agonist*) OR  ti: (glp 1 receptor agonist*) OR  ab: (glp 1 receptor agonist*) OR  ti: (glp 1 ra*) OR  ab: (glp 1 ra*) OR  ti: (glp 1ra*) OR  ab: (glp 1ra*) OR  ti: (glp 1ra*) OR  ab: (glp 1ra*) | 1,847 |
| **#4 (OAD 3)** | #2 AND #3 | 341 |
| **#5 (OAD 4)** | ti: (oha) OR  ab: (oha) OR  ti: (ogla) OR  ab: (ogla) OR  ti: (oad) OR  ab: (oad) OR  ti: (dpp 4 inhibitor*) OR  ab: (dpp 4 inhibitor*) OR  ti: (dpp iv inhibitor*) OR  ab: (dpp iv inhibitor*) OR  ti: (dpp4 inhibitor*) OR  ab: (dpp4 inhibitor*) OR  ti: (gliptin*) OR  ab: (gliptin*) OR  ti: (dipeptidyl peptidase iv inhibitor*) OR  ab: (dipeptidyl peptidase iv inhibitor*) OR  ti: (dipeptidyl peptidase 4 inhibitor*) OR  ab: (dipeptidyl peptidase 4 inhibitor*) OR  ti: (alogliptin*) OR  ab: (alogliptin*) OR  ti: (sulfonylurea*) OR  ab: (sulfonylurea*) OR  ti: (su) OR  ab: (su) OR  ti: (sodium glucose transporter 2 inhibitor*) OR  ab: (sodium glucose transporter 2 inhibitor*) OR  ti: (sodium glucose co transporter 2 inhibitor*) OR  ab: (sodium glucose co transporter 2 inhibitor*) OR  ti: (sodium glucose cotransporter 2 inhibitor*) OR  ab: (sodium glucose cotransporter 2 inhibitor*) OR  ti: (sglt 2 inhibitor*) OR  ab: (sglt 2 inhibitor*) OR  ti: (sglt2 inhibitor*) OR  ab: (sglt2 inhibitor*) OR  ti: (gliflozin*) OR  ab: (gliflozin*) OR  ti: (canagliflozin*) OR  ab: (canagliflozin*) OR  ti: (dapagliflozin*) OR  ab: (dapagliflozin*) OR  ti: (empagliflozin*) OR  ab: (empagliflozin*) OR  ti: (ertugliflozin*) OR  ab: (ertugliflozin*) OR  ti: (thiazolidinedione*) OR  ab: (thiazolidinedione*) OR  ti: (tzd*) OR  ab: (tzd*) OR  ti: (glitazone*) OR  ab: (glitazone*) | 9,649 |
| **#6 (OAD Total)** | #4 OR #5 | 9,936 |
| **#7 (Adherence)** | it: (Treatment Compliance) OR  ti: (adhere*) OR  ab: (adhere*) OR  ti: (non-adhere*) OR  ab: (non-adhere*) OR  ti: (nonadhere*) OR  ab: (nonadhere*) OR  ti: (complian*) OR  ab: (complian*) OR  ti: (noncomplian*) OR  ab: (noncomplian*) OR  ti: (non-complian*) OR  ab: (non-complian*) OR  ti: (comply*) OR  ab: (comply*) OR  ti: (noncomply*) OR  ab: (noncomply*) OR  ti: (non-comply*) OR  ab: (non-comply*) OR  ti: (persist*) OR  ab: (persist*) OR  ti: (nonpersist*) OR  ab: (nonpersist*) OR  ti: (non-persist*) OR  ab: (non-persist*) OR  ti: (discontin*) OR  ab: (discontin*) OR  ti: (concord*) OR  ab: (concord*) OR  ti: (omit*) OR  ab: (omit*) | 206,673 |
| **Combined** | #1 AND #6 AND #7 | 78 |
| **Date and language restrictions** | #8 AND Year: 2012 To 2022 | 54 |
| Date of Search | 12-JUL-2022 |  |

## Cochrane Trials

| Search | Search String | Results |
| --- | --- | --- |
| **#1 (T2D)** | [mh ^"diabetes mellitus, type 2"] OR  (Type 2 diabetes):ti,ab,kw OR  (Type 2 diabetes mellitus):ti,ab,kw OR  (Type ii diabetes):ti,ab,kw OR  (Type ii diabetes mellitus):ti,ab,kw OR  (T2D):ti,ab,kw OR  (T2DM):ti,ab,kw OR  (non-insulin dependent diabetes):ti,ab,kw OR  (NIDDM):ti,ab,kw OR  (Diabetes type 2):ti,ab,kw OR  (Diabetes type ii):ti,ab,kw OR  (Diabetes mellitus type 2):ti,ab,kw OR  (Diabetes mellitus type ii):ti,ab,kw OR  (PWT2D):ti,ab,kw | 55,025 |
| **#2 (OAD 1)** | (Oral):ti,ab,kw OR  (Orally):ti,ab,kw OR  [mh ^”administration, oral”] | 200,718 |
| **#3 (OAD 2)** | [mh "Hypoglycemic Agents"] OR  (hypoglycemic*):ti,ab,kw OR  (hypoglycaemic*):ti,ab,kw OR  (antidiabetic*):ti,ab,kw OR  (antihyperglycaemic*):ti,ab,kw OR  (antihyperglycemic*):ti,ab,kw OR  (glucose-lowering):ti,ab,kw OR  (glucagon like peptide 1 receptor agonist*):ti,ab,kw OR  (glp 1 receptor agonist*):ti,ab,kw OR  (glp 1 ra*):ti,ab,kw OR  (glp 1ra*):ti,ab,kw | 20,779 |
| **#4 (OAD 3)** | #2 AND #3 | 6,766 |
| **#5 (OAD 4)** | (OHA):ti,ab,kw OR  (OGLA):ti,ab,kw OR  (OAD):ti,ab,kw OR  [mh “Dipeptidyl-Peptidase IV Inhibitors"] OR  (dpp 4 inhibitor*):ti,ab,kw OR  (dpp 4 inhibitor*):ti,ab,kw OR  (dpp iv inhibitor*):ti,ab,kw OR  (dpp iv inhibitor*):ti,ab,kw OR  (dpp4 inhibitor*):ti,ab,kw OR  (gliptin*):ti,ab,kw OR  (dipeptidyl peptidase iv inhibitor*):ti,ab,kw OR  (dipeptidyl peptidase 4 inhibitor*):ti,ab,kw OR  (dipeptidyl peptidase iv inhibitor*):ti,ab,kw OR  (dipeptidyl peptidase 4 inhibitor*):ti,ab,kw OR  (dipeptidyl peptidase iv inhibitor*):ti,ab,kw OR  (dipeptidyl peptidase 4 inhibitor*):ti,ab,kw OR  (dipeptidyl peptidase iv inhibitor*):ti,ab,kw OR  (dipeptidyl peptidase 4 inhibitor*):ti,ab,kw OR  (alogliptin):ti,ab,kw OR  [mh “Sulfonylurea Compounds”] OR  (sulfonylurea*):ti,ab,kw OR  (SU):ti,ab,kw OR  [mh “Sodium-Glucose Transporter 2 Inhibitors”] OR  (sodium glucose transporter 2 inhibitor*):ti,ab,kw OR  (sodium glucose transporter 2 inhibitor*):ti,ab,kw OR  (sodium glucose co transporter 2 inhibitor*):ti,ab,kw OR  (sodium glucose co transporter 2 inhibitor*):ti,ab,kw OR  (sodium glucose transporter 2 inhibitor*):ti,ab,kw OR  (sodium glucose transporter 2 inhibitor*):ti,ab,kw OR  (sodium glucose co transporter 2 inhibitor*):ti,ab,kw OR  (sodium glucose co transporter 2 inhibitor*):ti,ab,kw OR  (sglt 2 inhibitor*):ti,ab,kw OR  (sglt2 inhibitor*):ti,ab,kw OR  (sglt 2 inhibitor*):ti,ab,kw OR  (gliflozin*):ti,ab,kw OR  [mh “canagliflozin”] OR  (canagliflozin*):ti,ab,kw OR  (dapagliflozin*):ti,ab,kw OR  (empagliflozin*):ti,ab,kw OR  (ertugliflozin*):ti,ab,kw OR  (thiazolidinedione*):ti,ab,kw OR  (tzd*):ti,ab,kw OR  (glitazone*):ti,ab,kw OR  [mh “Thiazolidinediones"] | 1,095,394 |
| **#6 (OAD Total)** | #4 OR #5 | 1,095,514 |
| **#7 (Adherence)** | [mh “Medication Adherence”] OR  (adhere*):ti,ab,kw OR  (non-adhere*):ti,ab,kw OR  (complian*):ti,ab,kw OR  (noncomplian*):ti,ab,kw OR  (comply*):ti,ab,kw OR  (noncomply*):ti,ab,kw OR  (persist*):ti,ab,kw OR  (nonpersist*):ti,ab,kw OR  (discontin*):ti,ab,kw OR  (concord*):ti,ab,kw OR  (omit*):ti,ab,kw | 171,726 |
| **#8 (Combined)** | #1 AND #6 AND #7 | 6,784 |
| **Date and language restrictions** | #8 with Publication Year from 2012 to 2022 | 4,842 |
| Date of Search | 12-JUL-2022 |  |

## Scopus

| Search | Search String | Results |
| --- | --- | --- |
| **#1 (T2D)** | TITLE-ABS-KEY(”type 2 diabetes”) OR  TITLE-ABS-KEY(”type 2 diabetes mellitus”) OR  TITLE-ABS-KEY(”type ii diabetes”) OR  TITLE-ABS-KEY(”type ii diabetes mellitus”) OR  TITLE-ABS-KEY(”t2d”) OR  TITLE-ABS-KEY(”t2dm”) OR  TITLE-ABS-KEY(”non-insulin dependent diabetes”) OR  TITLE-ABS-KEY(”niddm”) OR  TITLE-ABS-KEY(”diabetes type 2”) OR  TITLE-ABS-KEY(”diabetes type ii”) OR  TITLE-ABS-KEY(”diabetes mellitus type 2”) OR  TITLE-ABS-KEY(”diabetes mellitus type ii”) OR  TITLE-ABS-KEY(”pwt2d”) | 308,041 |
| **#2 (OAD 1)** | TITLE-ABS-KEY(”oral”) OR  TITLE-ABS-KEY(”orally”) | 1,358,129 |
| **#3 (OAD 2)** | TITLE-ABS-KEY(”hypoglycemic*”) OR  TITLE-ABS-KEY(”hypoglycaemic*”) OR  TITLE-ABS-KEY(”antidiabetic*”) OR  TITLE-ABS-KEY(”antihyperglycaemic*”) OR  TITLE-ABS-KEY(”antihyperglycemic*”) OR  TITLE-ABS-KEY(”glucose-lowering”) OR  TITLE-ABS-KEY(”glucagon like peptide 1 receptor agonist*”) OR  TITLE-ABS-KEY(”glp 1 receptor agonist*”) OR  TITLE-ABS-KEY(”glp 1 ra*”) OR  TITLE-ABS-KEY(”glp 1ra*”) | 155,740 |
| **#4 (OAD 3)** | #2 AND #3 | 44,907 |
| **#5 (OAD 4)** | TITLE-ABS-KEY(”oha”) OR  TITLE-ABS-KEY(”ogla”) OR  TITLE-ABS-KEY(”oad”) OR  TITLE-ABS-KEY(”dpp 4 inhibitor*”) OR  TITLE-ABS-KEY(”dpp iv inhibitor*”) OR  TITLE-ABS-KEY(”dpp4 inhibitor*”) OR  TITLE-ABS-KEY(”gliptin*”) OR  TITLE-ABS-KEY(”dipeptidyl peptidase iv inhibitor*”) OR  TITLE-ABS-KEY(”dipeptidyl peptidase 4 inhibitor*”) OR  TITLE-ABS-KEY(”alogliptin”) OR  TITLE-ABS-KEY(”sulfonylurea*”) OR  TITLE-ABS-KEY(”su”) OR  TITLE-ABS-KEY(”sodium glucose transporter 2 inhibitor*”) OR  TITLE-ABS-KEY(”sodium glucose co transporter 2 inhibitor*”) OR  TITLE-ABS-KEY(”sodium glucose cotransporter 2 inhibitor*”) OR  TITLE-ABS-KEY(”sglt 2 inhibitor*”) OR  TITLE-ABS-KEY(”sglt2 inhibitor*”) OR  TITLE-ABS-KEY(”gliflozin*”) OR  TITLE-ABS-KEY(”canagliflozin*”) OR  TITLE-ABS-KEY(”dapagliflozin*”) OR  TITLE-ABS-KEY(”empagliflozin*”) OR  TITLE-ABS-KEY(”ertugliflozin*”) OR  TITLE-ABS-KEY(”thiazolidinedione*”) OR  TITLE-ABS-KEY(”tzd*”) OR  TITLE-ABS-KEY(”glitazone*”) | 215,798 |
| **#6 (OAD Total)** | #4 OR #5 | 249,071 |
| **#7 (Adherence)** | TITLE-ABS-KEY(”adhere*”) OR  TITLE-ABS-KEY(”non-adhere*”) OR  TITLE-ABS-KEY(”nonadhere*”) OR  TITLE-ABS-KEY(”complian*'”) OR  TITLE-ABS-KEY(”noncomplian*”) OR  TITLE-ABS-KEY(”non-complian*”) OR  TITLE-ABS-KEY(”comply*”) OR  TITLE-ABS-KEY(”noncomply*”) OR  TITLE-ABS-KEY(”non-comply*”) OR  TITLE-ABS-KEY(”persist*”) OR  TITLE-ABS-KEY(”nonpersist*”) OR  TITLE-ABS-KEY(”non-persist*”) OR  TITLE-ABS-KEY(”discontin*”) OR  TITLE-ABS-KEY(”concord*”) OR  TITLE-ABS-KEY(”omit*”) | 1,843,207 |
| **#8 (Combined)** | #1 AND #6 AND #7 | 3,649 |
| **Date and language restrictions** | #8 AND (LIMIT-TO ( LANGUAGE , "English" ) ) AND ( LIMIT-TO ( PUBYEAR , 2022 ) OR LIMIT-TO ( PUBYEAR , 2021 ) OR LIMIT-TO ( PUBYEAR , 2020 ) OR LIMIT-TO ( PUBYEAR , 2019 ) OR LIMIT-TO ( PUBYEAR , 2018 ) OR LIMIT-TO ( PUBYEAR , 2017 ) OR LIMIT-TO ( PUBYEAR , 2016 ) OR LIMIT-TO ( PUBYEAR , 2015 ) OR LIMIT-TO ( PUBYEAR , 2014 ) OR LIMIT-TO ( PUBYEAR , 2013 ) OR LIMIT-TO ( PUBYEAR , 2012 ) ) | 2,272 |
| Date of Search | 13-JUL-2022 |  |
